# Distinct SUMO Ligases Cooperate with Esc2 and Slx5 to Suppress Duplication-Mediated Genome Rearrangements

**DOI:** 10.1371/journal.pgen.1003670

**Published:** 2013-08-01

**Authors:** Claudio P. Albuquerque, Guoliang Wang, Nancy S. Lee, Richard D. Kolodner, Christopher D. Putnam, Huilin Zhou

**Affiliations:** 1 Ludwig Institute for Cancer Research, University of California School of Medicine, San Diego, La Jolla, California, United States of America; 2 Department of Cellular and Molecular Medicine, University of California School of Medicine, San Diego, La Jolla, California, United States of America; 3 Moores-UCSD Cancer Center, University of California School of Medicine, San Diego, La Jolla, California, United States of America; The Scripps Research Institute, United States of America

## Abstract

Suppression of duplication-mediated gross chromosomal rearrangements (GCRs) is essential to maintain genome integrity in eukaryotes. Here we report that SUMO ligase Mms21 has a strong role in suppressing GCRs in *Saccharomyces cerevisiae*, while Siz1 and Siz2 have weaker and partially redundant roles. Understanding the functions of these enzymes has been hampered by a paucity of knowledge of their substrate specificity *in vivo*. Using a new quantitative SUMO-proteomics technology, we found that Siz1 and Siz2 redundantly control the abundances of most sumoylated substrates, while Mms21 more specifically regulates sumoylation of RNA polymerase-I and the SMC-family proteins. Interestingly, Esc2, a SUMO-like domain-containing protein, specifically promotes the accumulation of sumoylated Mms21-specific substrates and functions with Mms21 to suppress GCRs. On the other hand, the Slx5-Slx8 complex, a SUMO-targeted ubiquitin ligase, suppresses the accumulation of sumoylated Mms21-specific substrates. Thus, distinct SUMO ligases work in concert with Esc2 and Slx5-Slx8 to control substrate specificity and sumoylation homeostasis to prevent GCRs.

## Author Summary

The human genome contains many “at-risk” sequences that are prone to mutations, including diverse repeated sequences, segmental duplications and regions of copy number variations. Such repetitive sequence elements can cause genome rearrangements through non-allelic homologous recombination (NAHR) and many human diseases are caused by chromosomal rearrangements mediated by NAHR. Here we discovered that Mms21 dependent sumoylation has a major role in suppressing duplication-mediated GCRs. In addition, we showed that mutations of additional genes in sumoylation pathway cause the highest GCR defect known to date, demonstrating a critical role of sumoylation pathway in preventing duplication-mediated GCRs. We further developed a new quantitative proteomics technology to measure sumoylation levels of individual sumoylated proteins on a proteome-wide scale and applied this approach to uncover distinct and overlapping activities of SUMO ligases for substrate sumoylation. We further established the roles of Esc2 and Slx5 in regulating the SUMO proteome. Taken together these findings suggest a model in which a fine balance of Mms21 activity towards its substrates is critical for the suppression of chromosomal rearrangements. Our findings thus have important implications for cancer genetics as well as new insights into the regulation and substrate specificity of protein sumoylation enzymes.

## Introduction

The human genome contains many “at-risk” sequences that are prone to mutations including diverse repeated sequences, segmental duplications and regions of copy number variations [1], [2]. Such repetitive sequence elements can cause genome rearrangements through non-allelic homologous recombination (NAHR) and many human diseases are caused by chromosomal rearrangements mediated by NAHR [3]. Moreover, many cancers exhibit ongoing genome rearrangements, stimulated by numerous “at-risk” sequences in the genome. The yeast *Saccharomyces cerevisiae* provides a powerful model system to study genome rearrangements [4], and many genes in *S. cerevisiae* have been found to suppress gross chromosomal rearrangements (GCRs), including those mediated by single-copy sequences and those mediated by segmental duplications [5]. Interestingly, a number of these genes are involved in protein sumoylation [5]: Esc2 contains tandem SUMO-like domains [6]–[8], and Slx5 is a subunit of the Slx5-Slx8 complex, which was shown to be a SUMO-targeted ubiquitin ligase [9],[10]. In contrast, Siz1, a SUMO E3 ligase [11], does not have a significant role in suppressing duplication-mediated GCRs [12]. However, deletion of *SIZ1* suppresses the GCR defect of *asf1* mutant mediated by single-copy sequences to essentially wild-type levels [13]. Taken together, these observations raise the possibility that protein sumoylation may have a role in regulating and in some cases suppressing GCRs.

SUMO (small ubiquitin-like modifier) is a member of the ubiquitin-like protein family, and, like ubiquitin, is covalently attached by an isopeptide bond through its C-terminus to lysine residues of the target proteins via an enzymatic cascade [14]. The *SMT3* gene encodes SUMO in *S. cerevisiae*, and the Smt3 protein is activated by the Aos1-Uba2 complex, which is the *S. cerevisiae* SUMO E1 activating enzyme [15]. Smt3 is then transferred to Ubc9, the sole SUMO E2 enzyme in *S. cerevisiae* [16], which catalyzes sumoylation of target proteins. Although Ubc9 is capable of sumoylation by itself *in vitro*, substrate selectivity *in vivo* is generally controlled by SUMO E3 ligases. The three known *S. cerevisiae* SUMO E3 ligases in mitotic cells are Siz1, Siz2, and Mms21 [11], [17]. These SUMO E3 ligases are members of the PIAS (protein inhibitor of activated STAT) family and contain an SP-RING (Siz/PIAS RING) domain, which is similar to the RING domain in ubiquitin E3 ligases [14]. While Siz1 and Siz2 are single-polypeptide enzymes, Mms21 is a subunit of the multi-subunit Smc5-Smc6 complex [17]. Two SUMO-specific isopeptidases, Ulp1 and Ulp2, cleave SUMO from modified proteins [18]–[20]. Ulp2 is present throughout the nucleus, whereas Ulp1 is localized to the nuclear envelope through interactions with the nuclear pores [21], [22]. The non-redundant genes in the sumoylation pathway, *SMT3*, *AOS1*, *UBA2*, and *UBC9*, are all essential for viability in *S. cerevisiae*. In addition, *ULP1* and *MMS21* are also essential; however, the SUMO E3 ligase domain of *MMS21* is not required for cell viability [17]. Moreover, the SUMO-ligase deficient *mms21* mutation is lethal when it is combined with both *siz1* and *siz2* mutations, indicating a requirement of SUMO E3 ligases for sumoylation *in vivo* [23]. Protein sumoylation regulates numerous cellular processes, including protein transport, gene transcription, chromosome segregation, DNA repair, and meiosis [14], [24], [25]. The structure and enzymology of these sumoylation enzymes are relatively well understood; however, the identities of sumoylated substrates and specificity of the SUMO E3 ligases *in vivo* have been more poorly characterized, leaving a gap in our knowledge of the roles of sumoylation *in vivo*.

Several approaches have been taken to identify proteins sumoylated *in vivo*. A number of sumoylated proteins have been identified via a candidate-protein approach [23], [26]–[30]; however, this approach is difficult to apply to a proteome-wide study. On the other hand, mass spectrometry (MS) based methods have been used to more broadly identify sumoylated proteins in yeast [31]–[35]. In these studies, epitope-tagged SUMO was used for affinity-based purification of sumoylated proteins, which were either subjected to SDS-PAGE and in-gel digestion or liquid chromatography-based method for MS analysis. SDS-PAGE separation helps to remove unconjugated monomeric SUMO prior to MS analysis; however, this process limits the throughput and sensitivity of MS analysis [31]–[33]. In cases where liquid chromatography was used, the presence of the highly abundant Smt3 could compromise the detection of lower abundant sumoylated proteins [34], [35]. Importantly, varying levels of contaminant proteins are unavoidable during purification of sumoylated proteins. As a consequence, the lack of quantitative MS analysis to examine the levels of contaminant proteins in these previous studies limits the confidence that all of the proteins identified were actually sumoylated. Here we describe the development of a new proteomics technology for a quantitative analysis of protein sumoylation. Using stable isotope labeling method, we were able to exclude contaminant proteins and provide quantitative MS evidence for sumoylated proteins identified here. We then applied this new technology to determine the substrate specificity of SUMO E3 ligases Siz1, Siz2, and Mms21 on a proteome-wide scale for the first time. We further discovered a novel function of Esc2 in regulating the substrate selectivity of Mms21 and showed that the Slx5-Slx8 complex works in concert with Siz1, Siz2 and Mms21 to regulate substrate-specific sumoylation homeostasis *in vivo*. Together, these findings provide mechanistic insights into the functions of Esc2 and Slx5-Slx8 in suppressing GCRs and maintaining sumoylation homeostasis in conjunction with distinct SUMO ligases.

## Results

### Distinct SUMO ligases cooperate with Esc2 and Slx5-Slx8 to suppress duplication-mediated GCRs

To explore the roles of the SUMO E3 ligases in genome stability, we introduced deletions of *SIZ1* and *SIZ2* and two alleles of the essential *MMS21* gene, *mms21-11* and *mms21-CH*, into strains to measure the rates of accumulating GCRs. We tested the mutations in two strain backgrounds; strains containing the *yel068c::CAN1/URA3* assay monitor GCRs formed using single-copy sequences, whereas strains containing the *yel072w::CAN1/URA3* assay monitor GCRs formed both by a segmental duplication and by single-copy sequences (Table 1 and Figure S1) [5]. Neither the *siz1Δ* mutation nor the *siz2Δ* mutation caused an appreciable defect in suppressing GCR formed in either assay. In contrast, the *siz1Δ siz2Δ* double mutant strain had a synergistic increase in the rate of GCR formation specific to the duplication-mediated GCR assay. This is consistent with the increase in mitotic recombination (loss of heterozygosity) seen in the *siz1Δ siz2Δ* double mutant [36]. The *mms21-11* allele, which encodes a truncated Mms21 that lacks the SUMO ligase domain at the C-terminus [17], caused substantially increased GCR rates both in the *yel068c::CAN1/URA3* assay (∼47-fold), consistent with increases seen in a related GCR assay of the *smc6* mutant [37], and in the *yel072w::CAN1/URA3* assay (∼603-fold; Table 1). Combining the *mms21-11* allele with the *siz1Δ* mutation caused a 3-fold increase in the *yel068c::CAN1/URA3* assay and no increase in the *yel072w::CAN1/URA3* assay. In contrast, the *mms21-11 siz2Δ* double mutant strain had a ∼1,800-fold increase in the *yel072w::CAN1/URA3* assay relative to wild-type and a ∼3-fold increase relative to the *mms21-11* strain, which is the highest GCR rate yet reported with this assay [5], [12]. To verify that the increased GCR rates in *mms21-11* allele were due to loss of SUMO ligase activity, we also introduced the *mms21-CH* allele into the two GCR assay strains. The *mms21-CH* allele encodes an inactive SUMO ligase domain caused by alanine substitutions at the conserved Cys200 and His202 of the SP-RING domain [38]. The increases in GCR rates caused by the *mms21-CH* allele were very similar to *mms21-11*: (i) the single mutant strain caused a substantially increased GCR rate in both assays (∼9-fold and ∼215-fold for non-duplication mediated and duplication mediated assays, respectively), and (ii) combinations with deletions of *SIZ1* and *SIZ2* caused increased GCRs, with the largest effects being observed in the *yel072w::CAN1/URA3* assay and caused by additional deletion of *SIZ2* (Table 1). Taken together, these data indicate that the SUMO ligase activity of Mms21 suppresses GCRs mediated by single-copy sequences and plays an even more important role in suppressing GCRs mediated by segmental duplications. In contrast, Siz1 and Siz2 are partially redundant, consistent with previous studies [27], and relatively less important than Mms21 in suppressing GCRs. Additionally, Siz2 plays a relatively stronger role in suppressing GCRs than Siz1 in the absence of Mms21 SUMO ligase activity.

**Table 1:** Effect of mutation of SUMO E3 ligases, Esc2 and Slx5 on duplication-mediated GCRs.

Genotype	*yel068c::CAN1/URA3* GCR rate*	*yel072w::CAN1/URA3* GCR rate*	Ratio #
Wild type**	2.27×10^−9^ (1)	1.97×10^−8^ (8.7)	8.7
*siz1Δ* **	3.13×10^−10^ (0.1)	6.35×10^−8^ (28)	203
*siz2Δ*	2.66×10^−9^ (1.2)	3.71×10^−8^ (16.3)	14
*siz1Δ siz2Δ*	7.14×10^−9^ (3.1)	1.86×10^−7^ (82)	26
*mms21-11*	1.06×10^−7^ (46.7)	1.19×10^−5^ (5242)	112
*mms21-11 siz1Δ*	3.46×10^−7^ (152)	1.23×10^−5^ (5419)	36
*mms21-11 siz2Δ*	3.22×10^−7^ (142)	3.47×10^−5^ (15286)	108
*mms21-CH*	2.08×10^−8^ (9.2)	4.26×10^−6^ (1877)	205
*mms21-CH siz1Δ*	4.6×10^−8^ (20.3)	1.7×10^−5^ (7489)	369
*mms21-CH siz2Δ*	2.2×10^−7^ (97)	3.3×10^−5^ (14537)	150
*esc2Δ* ***	2.1×10^−8^ (9.3)	2.7×10^−6^ (1189)	128
*esc2Δ siz1Δ*	3.7×10^−8^ (16.3)	2.0×10^−6^ (881)	54
*esc2Δ siz2Δ*	1.9×10^−8^ (8.4)	1.7×10^−6^ (749)	89
*mms21-11 esc2Δ*	2.86×10^−7^ (126)	1.36×10^−5^ (5991)	48
*slx5Δ* **	1.48×10^−9^ (0.7)	4.8×10^−7^ (211)	324
*slx5Δ siz1Δ*	2.2×10^−8^ (9.7)	6.7×10^−7^ (295)	30
*slx5Δ siz2Δ*	9.5×10^−8^ (42)	4.5×10^−6^ (1982)	47
*slx5Δ siz1Δ siz2Δ*	9.9×10^−8^ (44)	8.4×10^−6^ (3700)	86

* Rate of accumulating Can 5-FOA progeny. Number in the parenthesis is the fold increase relative to wild-type *yel068c:CAN1/URA3* strain.

** Rates taken from [5], [12].

*** Rates of *esc2Δ* mutant are re-analyzed here.

# The *yel072w:CAN1/URA* rate divided by the *yel068c::CAN1/URA3* rate.

*ESC2* encodes a protein containing two SUMO-like domains [6]–[8] and plays a role in sister chromatid cohesion in conjunction with Smc5-Smc6 in the repair of damage during DNA replication [39], [40]. Deletion of *ESC2* also causes a strong defect in suppressing GCRs, particularly duplication-mediated GCRs, though less than that caused by the *mms21-11* allele (Table 1) [5]. Combining *esc2Δ* with *siz1Δ* or *siz2Δ* did not cause further increases in the GCR rates from either assay relative to the *esc2Δ*. The *esc2Δ mms21-11* double mutant strain had a similar GCR rate to the *mms21-11* rates in both GCR assays. Together these data suggest epistasis between *esc2Δ* and *mms21-11*, but cannot distinguish between an epistatic or additive effect of combining *esc2Δ* with *siz1Δ* and *siz2Δ* given the weak effects of the *siz1Δ* and *siz2Δ* mutations in the rate of accumulating GCRs.

Combining a deletion of *SLX5* with mutations of either *ESC2* or *MMS21* causes lethality [7], [29], suggesting *SLX5* functions in pathways that are redundant to *ESC2* and *MMS21*, and is potentially redundant with *SIZ1* and *SIZ2*. We therefore examined the genetic interactions between *SLX5*, *SIZ1* and *SIZ2*. The *slx5Δ* single mutation did not cause an increase in the *yel068c::CAN1/URA3* assay but did in the duplication-mediated *yel072w::CAN1/URA3* assay. Combining *slx5Δ* with *siz1Δ* or *siz2Δ* caused a synergistic increase in GCR rates in the *yel068c::CAN1/URA3* assay, and the *slx5Δ siz1Δ siz2Δ* triple mutant was not appreciably different than the *slx5Δ siz2Δ* double mutant. All of the *slx5Δ*, *siz1Δ*, and *siz2Δ* single mutations had stronger effects on the accumulation of GCRs in the duplication-mediated *yel072w::CAN1/URA3* assay (Table 1). Similarly, the combination of *slx5Δ* with *siz1Δ* and/or *siz2Δ* also caused a much greater effect on the accumulation of GCRs in the *yel072w::CAN1/URA3* assay than the *yel068c::CAN1/URA3* assay; however, the relative effects of the mutations was the same as in the *yel068c::CAN1/URA3* assay. Taken together the data indicate that *SLX5* functions redundantly with *SIZ1* and *SIZ2* to suppress GCRs, with *SIZ2* playing a more important role than *SIZ1* in the absence of *SLX5*.

### Quantitative and proteome-wide analysis identifies SUMO targets

**Figure 1:**
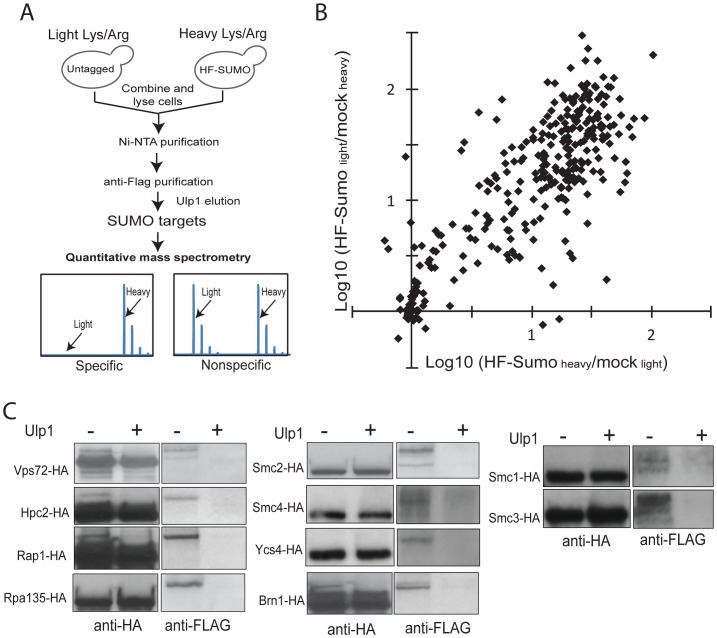
A new quantitative proteomics technology to characterize the SUMO-proteome. A) Method to identify and quantify sumoylated proteins using SILAC and MS. Untagged cells and HF-SUMO cells were combined for purification of sumoylated proteins. Ulp1 was used to elute sumoylated proteins for MS analysis. B) Scatter plot of the identified proteins based on two replicate experiments. The majority of the proteins show a large abundance ratio between HF-SUMO purification versus mock purification, indicating a highly purified sample. Candidate SUMO targets are identified based on at least 10-fold abundance ratio between HF-SUMO and mock sample in both replicate experiments. C) Western blot analysis of several SUMO targets confirmed them being sumoylated. Ulp1 treatment was used to treat half of the anti-HA immunoprecipitated sample.

In order to monitor sumoylation on a proteome-wide scale, we developed a quantitative proteomics method to identify and quantify sumoylated proteins (Figure 1A). We first integrated a 6×HIS-3×FLAG (HF) tag at the 5′-end of the *SMT3* gene at the chromosomal locus so that HF-SUMO and proteins covalently modified by HF-SUMO could be isolated by a tandem-affinity purification using Ni-NTA and anti-Flag columns. Since contaminant proteins are unavoidable and they could vary between independent purifications, we chose to metabolically label HF-SUMO cells and untagged cells using the Stable Isotope Labeling via the Amino acid in Cell culture (SILAC) method [41], [42]. An HF-SUMO tagged strain and an untagged strain that are both unable to synthesize lysine or arginine were grown in media that contained either ^14^N/^12^C-incorporated lysine and arginine or ^15^N/^13^C-incorporated lysine and arginine. Equal numbers of cells from both cultures were combined prior to cell lysis and purification of sumoylated proteins as one sample. This double labeling method then allowed us to monitor contaminant proteins during the purification of sumoylated proteins.

To remove the highly abundant unconjugated SUMO, which is a major barrier in the use of liquid chromatography-based proteomics technology for studying sumoylated proteins, we eluted the sumoylated proteins from the anti-Flag antibody column by specifically cleaving the isopeptide bonds between these proteins and HF-SUMO with Ulp1 [18]. The Ulp1-eluted proteins were then digested by trypsin and directly analyzed by multi-dimensional liquid chromatography and MS [43]. Proteins enriched in the HF-SUMO cells relative to the untagged cells, which are readily distinguished and quantified based on their isotope signatures, are candidate sumoylated proteins whereas those proteins with both isotope signatures are contaminant proteins that can be excluded from further analysis. We performed two replicate experiments with the HF-SUMO cells labeled by light lysine and arginine in the first experiment and by heavy lysine and arginine in the second experiment. We identified 653 proteins in the first and 695 proteins in the second experiment, and 306 proteins were identified in both replicates (Figure 1B). Among them, 176 proteins were strongly enriched (>10-fold) from HF-SUMO cells in both experiments (Table 2, Tables S1 and S2). Sites of modification could not be identified as Ulp1 treatment removes the SUMO modification; however, most targets were identified with fewer than 10 peptides and relatively low protein sequence coverage that would not be sufficient for SUMO site identification anyway (Tables S1 and S2). Our use of SILAC labeling, however, allows identification of sumoylated proteins at much higher sensitivities than methods dependent upon identification of sumoylation sites. We also compared the previously proteomic data sets and found a general lack of concordance between them (Figure S2A) [32]–[35]. Among them, the findings of Wohlschlegel *et al* and Denison *et al* share the most overlap with the targets identified here (Figure S2B), which were generally identified with more peptides per protein and likely represent the more abundant SUMO targets.

**Table 2:** A summary of 176 sumoylated proteins identified from two replicate MS experiments (see text for details).

Biological functions	SUMO targets identified
Septin-associated proteins	Cdc3, Cdc11, Shs1, Bud3, Bud4, Gin4, Hsl1, Kcc4, Bni5
Nuclear pore-associated	Mlp1, Mlp2, Nup2, Nup60
RNA Pol I associated proteins	Rpa135, Rpa190, Rpa43, (Rpo26), Uaf30, Reb1
RNA Pol II associated proteins	Rpb4, Rpo21, (Rpo26), Taf2, Taf3, Taf5, Taf12, Toa1, Nut1, Paf1, Spn1, Spt5, Spt15, Tfg1, Bdf1, Gcn5, Sgf73, Spt7
RNA Pol III associated proteins	Rpc37, Rpc53, Rpc82, (Rpo26), Ret1, Tfc3, Tfc4, Bdp1, Tfc6, Tfc7, Brf1
Gene-specific transcription factors	Azf1, Bur6, Cbf1, Cin5, Tup1, Cyc8, Sko1, Cti6, Crz1, Cst6, Dig1, Ste12, Tec1, Gcr1, Gcr2, Hap1, Hms1, Met4, Mot1, Ngg1, Sef1, Sum1, Swi4, Tye7, Upc2, Vhr1, Vhr2, Wtm1
mRNA processing	Cet1, Hrp1, Lhp1, Prp45, Rrp5, Spp41, Stb3, Sub2
Chromatin associated proteins	Hir2, Hpc2, Hmo1, H2A, H2B, Isw1, Isw2, Itc1, Pob3, Sin3, Vid21, Rsc1, Rsc2, Rsc6, Rsc8, Rsc58, Npl6, Sth1, Rtt102, Rvb1, Rvb2, Snf2, Snf5, Swi3, Swr1, Swc3, Swc5, Vps72
Telomere, rDNA and gene silencing	Asf2, Ebp2, Fob1, Kre33, Net1, Rap1, Rif1, Sir3, Sir4, Tof2
Cohesin and condensin	Smc1, Smc3, Mcd1, Smc2, Smc4, Ycs4, Ycg1, Brn1, Smc5, Smc6
Chromosome segregation	Bir1, Cbf2, Fin1, Mad1, Sli15, Slk19, Spc24, Stu1
DNA replication and repair	Abf1, Orc3, Mcm6, Mrc1, Pol12, Pol30, Top1, Top2, Saw1, Rad16
SUMO enzymes	Aos1, Uba2, Ubc9, Siz1, Siz2
Other processes	Abp1, Bop3, Cdc48, Crn1, Ede1, Eft2, Eno2, Hsp104, Ipp1, Mrp8, Nba1, Ola1, Paa1, Pdc1, Pgk1, Ris1, Scs2, Spa2, Tal1, Tif2, Tkl1, Zeo1, Ymr111c

Some proteins have multiple functions but are classified in only one of their functions for simplicity.

We selected 10 candidate proteins identified above to verify the presence of the SUMO modification in unperturbed cells, including Vps72, Hpc2, Rap1, Rpa135, subunits of cohesin (Smc1 and Smc3), and subunits of condensin (Smc2, Smc4, Ycs4, and Brn1). For each protein of interest, we integrated a 3×HA tag at 3′-end of the encoding gene at the endogenous locus in the HF-SUMO strain. We then lysed cultures and purified the tagged protein via anti-HA immunoprecipitation. Part of the immunoprecipitate was treated by recombinant Ulp1, and treated and untreated immunopreciptates were analyzed by anti-HA and anti-FLAG Western blots (Figure 1C). The anti-FLAG antibody, which recognizes HF-SUMO, detected a species that was eliminated by Ulp1 treatment. This anti-FLAG reactive species had a higher molecular weight than the species recognized by the anti-HA antibody, which primarily detects the unmodified protein. Thus this analysis indicated both that all of these candidate proteins are sumoylated, and that only a fraction of the endogenous protein is sumoylated under non-perturbed conditions.

We classified the SUMO targets identified by MS according to their known functions (Table 2). Consistent with previous findings [23], [26], [30], [44]–[47], Septins, PCNA and many known sumoylated proteins are identified. Despite the differences between previously proteomic studies and our study here [32]–[35], which could be attributed to the methods used, considerable overlaps in the identified proteins are found (Figure S2). By far, most SUMO targets identified are involved in gene expression, including the core subunits of RNA polymerases, their associated proteins, chromatin remodelers, numerous gene-specific transcription factors and proteins involved in mRNA processing and transport. Of particular interests here are the proteins involved in DNA replication and repair. We observed fewer DNA repair proteins than have been reported [26], likely due to the fact that the cells studied here were not exposed to DNA damaging treatments. It is also interesting to note that many proteins that function in chromosomal organization were found as sumoylated substrates, including cohesin (Smc1, Smc3, Mcd1), condensin (Smc2, Smc4, Brn1, Ycs4, Ycg1) and the Smc5-Smc6 complex. In summary, the majority of the SUMO targets identified here function in the nucleus; consistent with a diverse role of protein sumoylation in nuclear processes.

We also often identified multiple subunits of large protein complexes (Table 2). This is likely to be due to multiple subunits being sumoylated and unlikely to be due to co-purification. We used strong denaturing conditions during the purification (see Experimental procedures). Additionally, combining untagged cells and HF-SUMO cells prior to their lysis for subsequent purification would exclude subunits undergoing rapid exchange, as peptides from these subunits would be identified with both light and heavy isotope signatures. In the case of septin sumoylation, we identified the known sumoylation targets Cdc3, Cdc11, and Shs1, but not the Cdc10 or Cdc12 subunits that are not known to be modified [44]. Similarly, for most of the complexes for which we identified multiple subunits, we consistently identified the same subsets of the complex in multiple subsequent experiments. Indeed, sumoylation of multiple subunits of a protein complex often occurs [26], [44], [48]. This could be due to either promiscuous sumoylation *in vivo*, or alternatively redundancy and/or cooperativity during signaling in downstream events such as the recruitment of proteins via SUMO-interaction motifs [49], [50].

### Specificity of the Siz1, Siz2 and Mms21 E3 SUMO ligases

We next sought to determine the specificity of the SUMO E3 ligases Siz1, Siz2 and Mms21. We therefore compared the relative abundance of each SUMO target in wild-type strains and various strains with defects in specific E3 SUMO ligases using quantitative MS. A wild-type strain expressing HF-SUMO and a *siz1Δ*, *siz2Δ*, *mms21-11*, or *siz1Δ siz2Δ* strain expressing HF-SUMO were grown in media that labeled proteins with arginines and lysines containing ^15^N/^13^C or ^14^N/^12^C, respectively. Equal numbers of cells from the wild-type strain and a mutant strain were then mixed and lysed, and the SUMO-modified proteins were isolated by tandem-affinity purification and Ulp1-elution. The eluent was then digested by trypsin, and peptides identified by tandem MS. Changes in the levels of sumoylation of targets were determined by calculating the ratio of intensities of individual peptides from the mutant strains relative to the intensities from the wild-type strain (Tables S3, S4, S5, S6).

**Figure 2:**
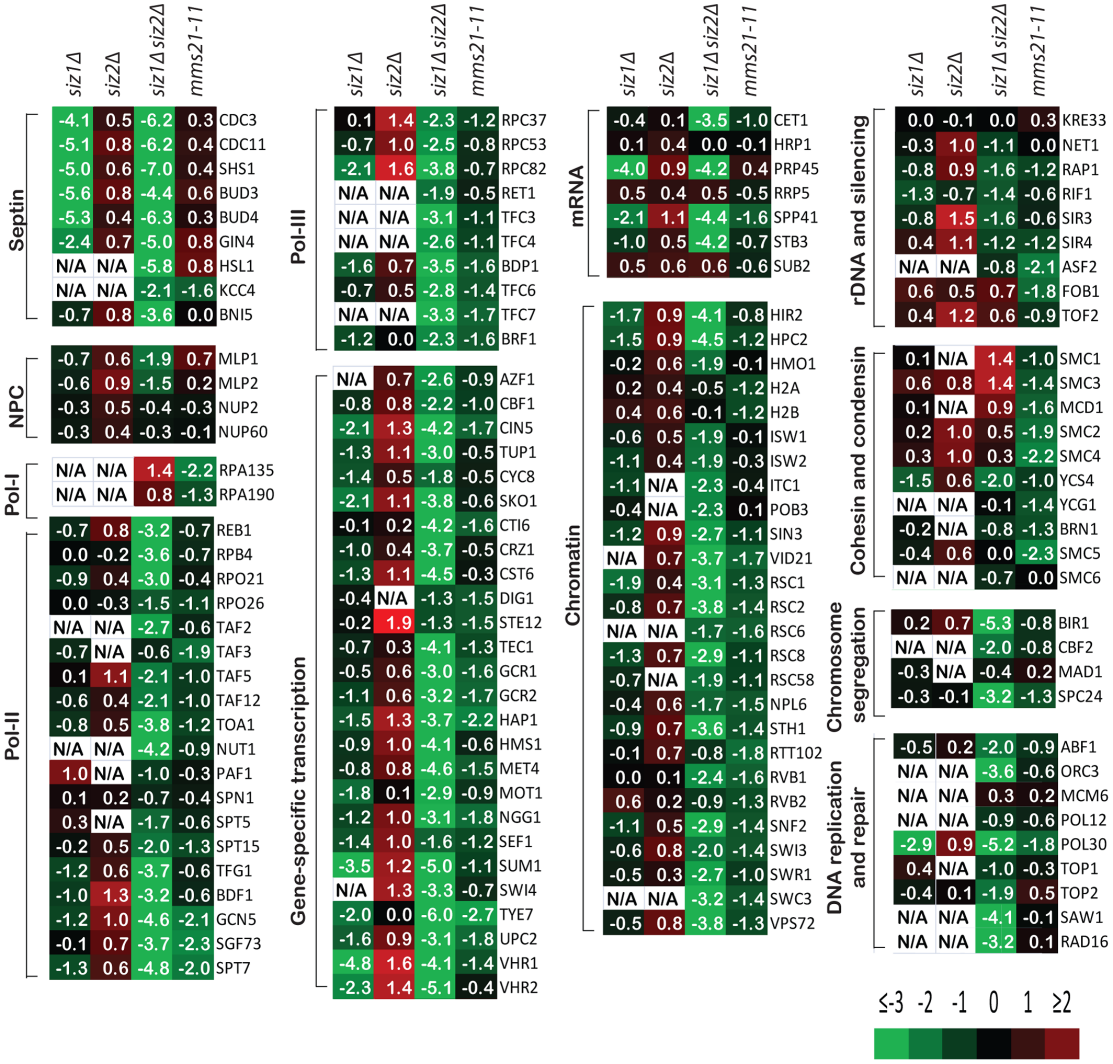
A summary of the effect of SUMO ligase-null mutations on the abundance of SUMO targets as measured by quantitative MS. Data are shown in the same order of *siz1Δ*, *siz2Δ*, *siz1Δ siz2Δ* and *mms21-11* mutants from left to right for each SUMO target. Log2 scale was used to calculate the relative abundance ratios. Occasionally, some SUMO targets were not identified and are indicated as not available (N/A). Additional information can be found in Tables S3, S4, S5, S6.

Remarkably, sumoylation of only a few proteins was entirely or almost entirely dependent upon Siz1 (Figure 2). These included the septins Cdc3, Cdc11 and Shs1, as well as their associated proteins Bud3 and Bud4, in agreement with previous findings [11]. Other Siz1-specific targets were also identified, including the pre-mRNA splicing factor Prp45, the transcription factors Sum1 and Vhr1, and the proteins of unknown function Bop3 and Mrp8 (Table S3). In contrast, none of the SUMO targets was specific to Siz2 exclusively. Unexpectedly, the major effect of the *siz2Δ* mutation was a modest increase in the abundance of many SUMO targets compared to wild-type cells, which could possibly be caused by up-regulation of Siz1 and/or Mms21 activity in the *siz2Δ* strain. This up-regulation is unlikely to be due to delays in S-phase as *siz2Δ* was not identified in screen of mutation altering the *S. cerevisiae* replication timing program [51]. For the *siz1Δ siz2Δ* double mutant strain, we observed a strong reduction of sumoylation of the majority of SUMO targets, indicating that Siz1 and Siz2 mediate sumoylation of many of the same targets and that biochemical redundancy explains the genetic interactions between *SIZ1* and *SIZ2* in suppressing genomic instability (Table 1). Sumoylated proteins primarily dependent upon either Siz1 or Siz2 for sumoylation included Pol30/PCNA, which had undetectable sumoylation in the *siz1Δ siz2Δ* double mutant strain as previously reported [52]. In addition, we identified proteins involved in chromosome segregation (Bir1, Cbf2, and Spc24), DNA replication (Abf1 and Orc3), DNA repair (Saw1 and Rad16), RNA polymerase II transcription (Rpo21, Rpo26, Rpb4, Reb1, Toa1, and subunits of TFIIA, TFIID, TFIIG, and SAGA), and RNA polymerase III transcription (Rpc37, Rpc53, Rpc82, Rpo26, and subunits of TFIIIB and TFIIIC). In contrast, the amounts of RNA polymerase I specific subunits Rpa135 and Rpa190 were higher in the *siz1Δ siz2Δ* double mutant strain compared to the wild-type strain. Thus, Siz1 and Siz2 have a highly redundant role in the sumoylation of proteins involved in gene transcription, chromatin remodeling and others with the notable exception of RNA polymerase I subunits such as Rpa135.

We next examined the effect of *mms21-11* mutation on the abundance of sumoylated proteins (Figure 2). In contrast to the *siz1Δ siz2Δ* mutant strain, most SUMO targets only underwent a modest decrease in sumoylation, including some of the RNA polymerase II and III related proteins and proteins involved in chromatin maintenance and remodeling. Sumoylated targets that were decreased primarily in the *mms21-11* strain but not the *siz1Δ siz2Δ* double mutant strain included the RNA polymerase I subunit Rpa135 and the structural maintenance of chromosome (SMC) family of proteins, Smc1, Smc2, Smc3, Smc4 and Smc5. In summary, the *siz1Δ*, *siz2Δ* and *mms21-11* mutations cause distinct perturbations to a subset of SUMO targets with Siz1 and Siz2 having redundant roles for the majority of proteins sumoylated in unperturbed cells.

### Sumoylation of RNA polymerase I and the SMC-family proteins requires Mms21

**Figure 3:**
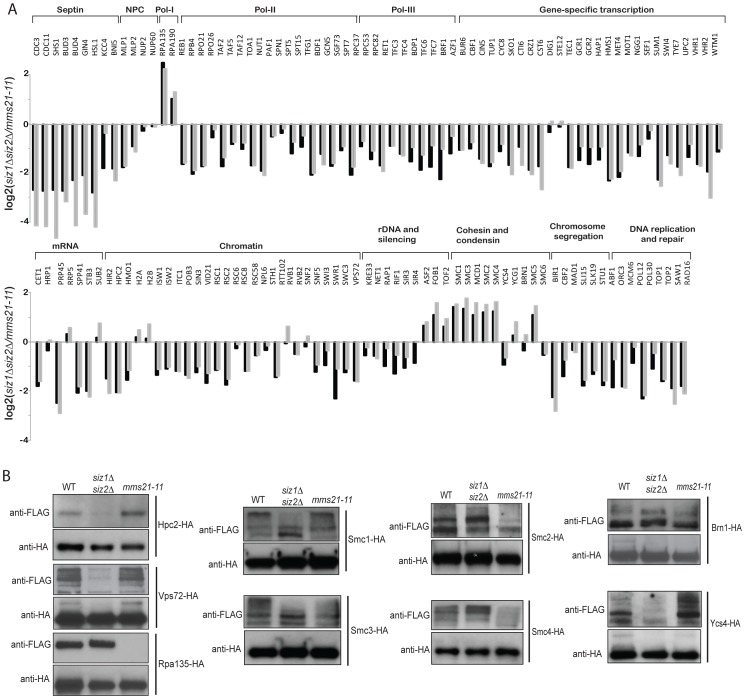
Identification of SUMO targets that are more specific to Mms21 than Siz1 and Siz2. A) Grey bar shows the calculated ratio between Mms21-dependent ratio and Siz1/Siz2 dependent ratio, using the data shown in Figure 2. Black bar shows a direct comparison between *mms21-11* and *siz1Δ siz2Δ* mutants using quantitative MS. B) Effects of *mm21-11* and *siz1Δ siz2Δ* on the sumoylation of selected SUMO targets. Each SUMO target was purified by anti-HA antibody and probed for its sumoylation using anti-FLAG Western blot.

Since the defects in suppression of GCRs caused by the tested *mms21* hypomorphic alleles were stronger than that caused by the *siz1Δ siz2Δ* double mutation (Table 1), we sought to gain insight into Mms21-specific substrates. First, we calculated the ratio between Mms21-dependent ratio (*mms21-11* versus wild-type) and Siz1/Siz2 dependent ratio (*siz1Δ siz2Δ* versus wild-type) for each SUMO target, which is equivalent to the abundance ratio between *mms21-11* and *siz1Δ siz2Δ* mutants. Most SUMO targets were more specific to Siz1/Siz2 than Mms21 (Figure 3A, grey bars). For example, the amounts of septin-associated proteins are much higher in *mms21-11* mutant compared to *siz1Δ siz2Δ* mutant. Similarly, numerous transcription-related proteins are more specific to Siz1/Siz2 than to Mms21. Interestingly, we observed a small subset of proteins that were less sumoylated in the *mms21-11* mutant strain compared to the *siz1Δ siz2Δ* double mutant strain. These proteins included RNA polymerase I, the ribosomal DNA-associated proteins Fob1 and Tof2, and the SMC-family of cohesin and condensin subunits. Second, we confirmed these findings experimentally by using quantitative MS to compare the abundance of sumoylated proteins in the *mms21-11* mutant strain with the *siz1Δ siz2Δ* double mutant strain (Figure 3A, black bars; Table S7). The results were essentially identical to the previously calculated ratio of ratios, which indicated the accuracy and quality of the MS data in three independent MS experiments (*siz1Δ siz2Δ* versus wild-type, *mms21-11* versus wild-type, and *mms21-11* versus *siz1Δ siz2Δ*). The robustness is due, in part, to the use of multiple peptides for protein quantification that allow changes as small as two-folds to be accurately measured.

To further substantiate the MS findings, we examined sumoylation of several SUMO targets using Western blot analysis. Among Siz1/Siz2-specific targets, sumoylation of Hpc2 and Vps72 were drastically reduced in the *siz1Δ siz2Δ* double mutant strain but not the *mms21-11* mutant strain (Figure 3B), in agreement with the MS findings. Also consistent with the MS results, sumoylation of Rpa135 was not detected in the *mms21-11* mutant strain but was unaffected in the *siz1Δ siz2Δ* double mutant strain (Figure 3B). On the other hand, sumoylation of the cohesin and condensin subunits had a more complex dependency on Siz1, Siz2 and Mms21. In contrast to a previous report [47], sumoylation of Smc1 and Smc3 of the cohesin complex was modestly reduced by the *mms21-11* mutation (Figure 3B), consistent with our MS results (Figure 2). The condensin subunits Smc2 and Smc4 had a stronger reduction of sumoylation levels in the *mms21-11* mutant strain than the *siz1Δ siz2Δ* double mutant strain (Figure 3B); however, sumoylation of the condensin subunit Ycs4 was more strongly reduced in the *siz1Δ siz2Δ* mutant strain, while sumoylation of the condensin subunit Brn1 was not significantly affected in either the *mms21-11* or the *siz1Δ siz2Δ* mutant strains. These observations indicate that Mms21 has a relatively stronger role in the sumoylation of the SMC subunits of cohesin and condensin than Siz1 and Siz2. Moreover, sumoylation of RNA polymerase I subunit Rpa135 is almost entirely dependent on Mms21.

### Esc2 regulates sumoylation of Mms21-preferred substrates

**Figure 4:**
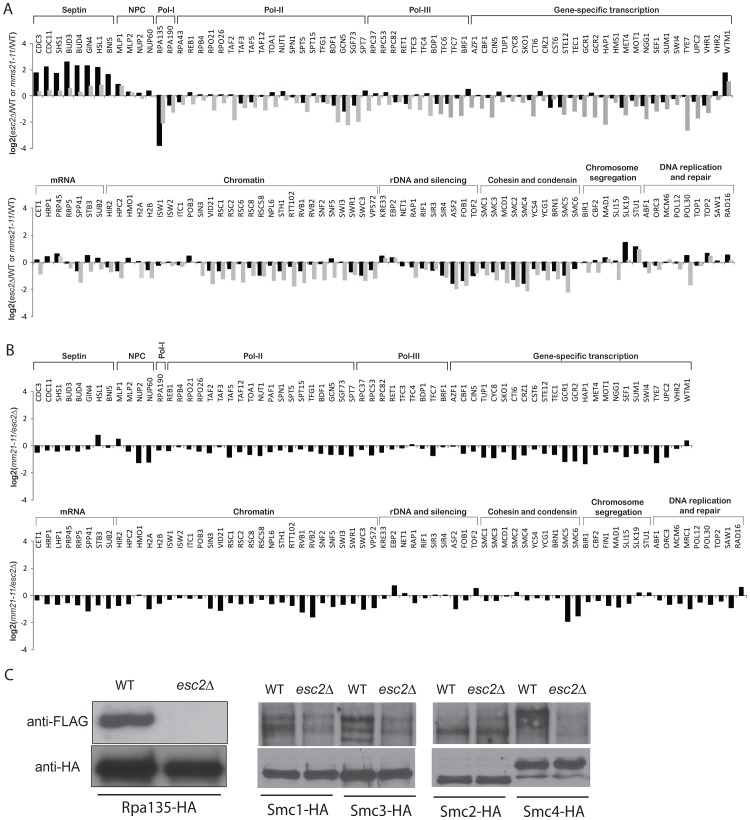
Effect of *esc2Δ* on the sumoylation of SUMO targets. A) Bar graph shows the relative abundance of SUMO targets between *esc2Δ* and wild-type cells (black bars). As a comparison, the results of *mms21-11* versus wild-type cells are shown in grey bars. B) Quantification of the relative abundance of SUMO targets between *esc2Δ* and *mms21-11* mutants. C) Western blot analysis of the effect of *esc2Δ* on the sumoylation of SMC proteins and Rpa135. Each SUMO target was purified by anti-HA antibody and probed for its sumoylation by anti-FLAG Western blot.

Esc2 has been shown to interact with Ubc9 and SUMO itself [7], and the *ESC2* deletion was found to be epistatic to the *mms21-11* mutation (Table 1). Thus, Esc2 might play a role in promoting the sumoylation of Mms21 substrates. To test this, we first used quantitative MS to determine the abundance ratios of sumoylated proteins between the wild-type strain and the *esc2Δ* mutant strain (Figure 4A, black bars; Table S8). Comparison with the abundance ratios of sumoylated proteins between the wild-type strain and the *mms21-11* mutant strain (Figure 4A, grey bars) revealed an overall similarity between the effects of *esc2Δ* and *mms21-11* on the abundances of most SUMO targets. For example, sumoylation of septins was elevated in both *esc2Δ* and *mms21-11* mutant strains compared to the wild-type strain, while that of Rpa135, Smc2 and Smc4 was reduced in both *esc2Δ* and *mms21-11* mutants. When we verified this similarity through direct comparison of the abundance of sumoylated proteins in the *esc2Δ* and the *mms21-11* mutant strains by quantitative MS, we found little change in the abundances of most SUMO targets between *esc2Δ* and *mms21-11* mutants (Figure 4B, Table S9). Together, these data indicate that Esc2 and Mms21 have similar roles on the sumoylated proteins in cells and are consistent with previously observed genetic epistasis in maintaining genome stability (Table 1).

We also determined the relative specificity between Esc2 and Siz1/Siz2 for sumoylation of targets by calculating the ratio of the *esc2Δ*/wild-type (Figure 4A) and *siz1Δ siz2Δ*/wild-type (Figure 2) relative abundance ratios (Figure S3). Most SUMO targets were more strongly dependent on Siz1/Siz2 than Esc2; however, a small subset of SUMO targets was more specific to Esc2, including Rpa135 and the SMC subunits of cohesin and condensin, similar to the comparison between the *mms21-11* and *siz1Δ siz2Δ* strains (Figure 3A). We confirmed these MS findings by Western blot analysis and found that sumoylation of Rpa135 was not detectable in the *esc2Δ* mutant (Figure 4C), similar to *mms21-11* (Figure 3B). Sumoylation of Smc1, Smc2 and Smc3 were modestly reduced by *esc2Δ*, and sumoylation of Smc4 was more strongly reduced by *esc2Δ*. In summary, Esc2 and Mms21 had a similar, although not identical, effect on the sumoylation levels of most SUMO targets *in vivo*. Both Esc2 and Mms21 exhibited stronger specificity towards sumoylation of Rpa135 and the SMC-family subunits of cohesin and condensin such as Smc4.

### Slx5 antagonizes sumoylation of Mms21-specific targets

**Figure 5:**
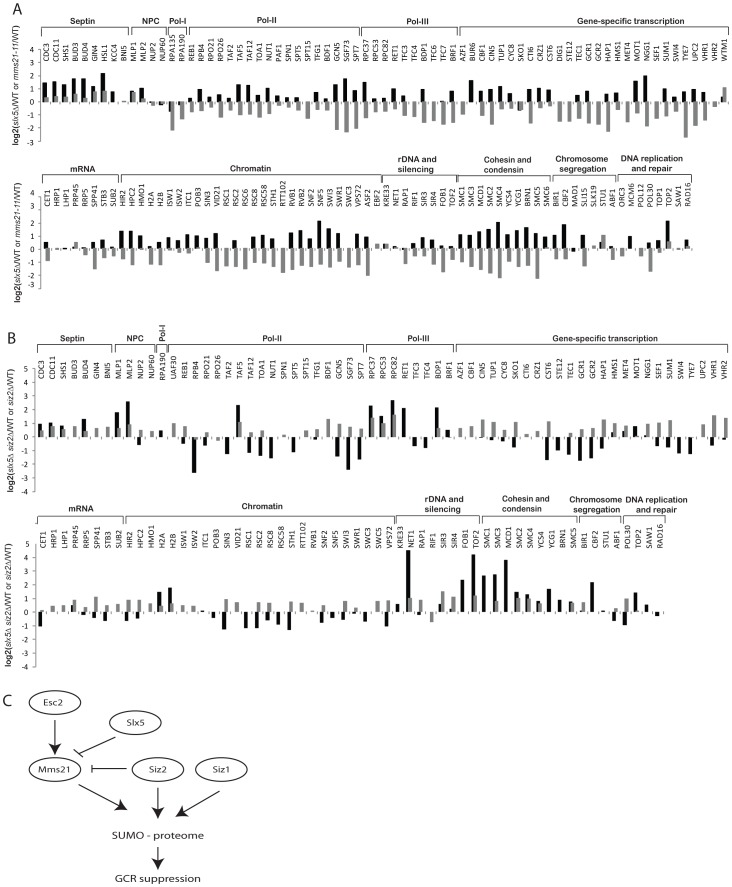
Effect of *slx5Δ* on the sumoylation of SUMO targets. A) Bar graph shows the relative abundance of SUMO targets between *slx5Δ* and wild-type cells (black bars). Results of *mms21-11* versus wild-type are included for comparison (grey bars). B) Bar graph shows the relative abundance of SUMO targets between *slx5Δ siz2Δ* and wild-type cells (black bars). Results of *siz2Δ* versus wild-type are included for comparison (grey bars). C) A proposed model of the regulation of sumoylation homeostasis in cells. Mms21-specific sumoylation is positively regulated by Esc2 and negatively regulated by Slx5 to achieve a balance of sumoylation in cells, which is critical to suppress GCRs.

Slx5 and its mammalian ortholog Rnf4 bind to sumoylated proteins and promote ubiquitination events [10], [53], [54]. To determine the role of Slx5-Slx8 complex in regulating the SUMO proteome, we compared the abundances of sumoylated proteins in the wild-type strain and the *slx5Δ* mutant strain using quantitative MS (Table S10). The net effect of deleting *SLX5* was to increase the overall abundance of most of the sumoylated proteins (Figure 5A, black bars). Siz1-specific targets were only weakly affected; sumoylation of septins was modestly increased, whereas sumoylation of Pol30/PCNA, Sum1 and Prp45 was not appreciably altered. In contrast, sumoylation of Mms21-specific targets were elevated, including cohesin and condensin subunits; sumoylation of Smc4 increased by 4-fold. Together, these data suggested that Slx5 more specifically suppressed the accumulation of sumoylated Mms21 targets. Since *mms21-11* had a broad effect on many SUMO targets (Figure 2), we compared the abundance ratios of *slx5Δ*/wild-type and *mms21-11*/wild-type and observed an inverse correlation between them with the exceptions of septins and RNA polymerase I (Figure 5A, compare black and grey bars). The sumoylation events down-regulated in the *mms21-11* mutant strain, such as cohesin and condensin subunits, were up-regulated in the *slx5Δ* mutant strain, indicating an antagonistic relationship between Slx5 and Mms21.

Next, we examined the effect of deleting *SIZ1* on the SUMO proteome in *slx5Δ* cells. As expected, there was a drastic reduction of sumoylation of Siz1-specific targets including septins and Pol30 (Figure S4, Table S11). Overall, the effect of deleting *SIZ1* on the SUMO proteome of the *slx5Δ* mutant strain was similar to that of deleting *SIZ1* in the wild-type strain, indicating a lack of functional interaction between *SIZ1* and *SLX5*. In contrast, deletion of *SIZ2*, which alone caused a modest increase in sumoylated proteins (Figure 2), caused a further increase in sumoylation of cohesion and condensin subunits when combined with deletion of *SLX5* (Figure 5B, Table S12). As expected, Siz1-specific targets such as septins and Pol30 were not appreciably affected in the *siz2Δ slx5Δ* mutant. Thus, the deletion of *SLX5* increases sumoylation of Mms21-specific targets such as cohesin and condensin subunits, which is further exacerbated by deletion of *SIZ2*.

## Discussion

### Mms21, Siz1, and Siz2 have overlapping and distinct substrate specificities

Protein sumoylation is an important post-translational modification that regulates many cellular processes including, protein transport, gene transcription, and DNA repair [14], [25], [55]. Genetic studies have suggested that the three E3 SUMO ligases in *S. cerevisiae*, Mms21, Siz1, and Siz2, have distinct as well as overlapping functions [17], [56], [57]. Although the E3 SUMO ligases for a few SUMO substrates were known [23], [45], [47], here we have comprehensively analyzed the proteome-wide substrate specificities of Siz1, Siz2, and Mms21 in *S. cerevisiae* using a novel quantitative proteomics strategy. Several key findings are summarized here.

First, Siz1 and Mms21 specifically sumoylate a subset of substrates, whereas we could not detect any Siz2-specific SUMO targets in unperturbed cells. Siz1 plays a major role in the sumoylation of septin subunits and septin-associated proteins as well as some factors involved in RNA polymerase II transcription. In contrast, Mms21 plays an important role in sumoylation of proteins in the nucleolus, including RNA polymerase I, Fob1, and Tof2, as well as cohesin and condensin subunits that play roles both in the nucleolus and the nucleus. Lack of Siz2-specific targets may be due to the lower abundance of Siz2-specific targets in unperturbed cells, to a specific role of Siz2 during certain stresses [48], or to redundancy between *SIZ2* and other E3 SUMO ligases. In the *siz1Δ siz2Δ* double mutant strain, sumoylation of the majority of SUMO targets was down regulated. This result indicates that there is a strong overlap in the substrate specificity between Siz1 and Siz2, which is likely due to the fact that the *SIZ1* and *SIZ2* genes are paralogs arising from the whole genome duplication event that occurred in the ancestor of *S. cerevisiae* and related yeast species [58].

Second, multiple subunits of the same protein complex are often sumoylated. As an example, Smc2, Smc4, Ycs4 and Brn1 of condensin are found to be sumoylated. Interestingly, although Mms21 preferentially regulates sumoylation of Smc2 and Smc4, Siz1 and Siz2 are more important for the sumoylation of Ycs4. Moreover, both Siz1/Siz2 and Mms21 control sumoylation of Brn1. This observation argues against promiscuous sumoylation catalyzed by any single SUMO ligase *in vivo*. Instead, it raises the possibility that distinct sumoylation of different subunits of the same protein complex may lead to different biological outcomes.

Third, our MS data indicate that a balance of activities from Siz1, Siz2 and Mms21 controls the abundances of SUMO targets in cells. For example, *siz2Δ* causes increased sumoylation of many SUMO targets (Figure 2), which may be due to elevated activities of Siz1 and/or Mms21. Similarly; *siz1Δ siz2Δ* causes increased sumoylation of several SMC-family of proteins, consistent with up regulation of Mms21 activity. It should also be noted here that despite the importance of Mms21 for sumoylating the SMC-family proteins, Siz1 and Siz2 still contribute to their sumoylation to a lesser extent (Figure 3). Since MS only detects the levels of sumoylated proteins between WT and SUMO ligase mutants, we cannot exclude the possibility that the observed change of sumoylation could be due to its protein abundance change caused by mutations of SUMO ligases, which should be evaluated by alternative methods during further studies.

### Esc2 is a regulator of Mms21 activity *in vivo*

Mms21 is part of the multi-subunit Smc5-Smc6 complex [17]. Little was known about how Mms21 achieves its substrate selectivity. Genetic analyses suggest that Esc2 may function with Mms21. Both *MMS21* and *ESC2* suppress duplication-mediated GCRs (Table 1), are epistatic with each other (Table 1), and prevent the formation of X-shaped DNA replication intermediates [7], [39], [40]. Esc2 contains SUMO-like domains and no known enzymatic activity [6], but physically interacts with components of the SUMO pathway, including Ubc9 and Smt3 itself [7]. Previous studies of Rad60, an ortholog of Esc2 in fission yeast, identified an interaction between Rad60 and the Smc5-Smc6 complex and showed that Rad60 functions in the same pathway as Nse2, the fission ortholog of Mms21 [59], [60]. Here we found that *esc2Δ* has a similar but not identical effect as *mms21-11* on the sumoylation of most SUMO targets (Figure 4), indicating that Esc2 positively regulates Mms21 activity *in vivo* (Figure 5C), which is consistent with their epistatic relationship and the physical interactions between *S. pombe* Rad60 with Smc5-Smc6 and Ubc9 (Table 1) [59], [60]. Taken together, these data would be consistent with a role of Esc2 in promoting the formation of an active complex between Ubc9 and the Smc5-Smc6-Mms21 complex.

### Slx5 and Siz2 suppress sumoylation of Mms21-specific substrates

The Slx5-Slx8 complex has been implicated in protein sumoylation pathway [29]. Studies of Slx5 in yeasts and its ortholog Rnf4 in mammalian cells have shown that its role to the SUMO pathway is related to the SUMO-interacting motifs (SIMs) of Slx5/Rnf4, which bind to sumoylated proteins to catalyze ubiquitination events [10], [53], [54], and these data have been used to suggest that Slx5-Slx8 regulates the abundance of sumoylated proteins in cells. In addition, poly-sumoylation was suggested to cause an *slx8Δ* defect in *S. pombe* [59]; paradoxically poly-sumoylation could also suppress *slx5Δ* phenotype in *S. cerevisiae* [61]. While we do not have information on the effect of *slx5Δ* on poly-sumoylation, we found that Slx5 has a specific and novel role in suppressing sumoylation of Mms21-specific substrates. Our quantitative MS results cannot distinguish between mechanisms in which Slx5-Slx8 antagonizes Mms21 by ubiquitination and degradation of its sumoylated targets, which is consistent with its known ubiquitin ligase and SUMO-binding activities, from mechanisms by which Slx5-Slx8 antagonizes by directly or indirectly preventing Mms21 from sumoylating its targets. Deletion of *SIZ2* causes a modestly higher sumoylation of Mms21-specific targets, such as SMC-family proteins, and this increase in the accumulation of sumoylated targets is further elevated in the *siz2Δ slx5Δ* mutant strain (Figure 5B). As Siz2 mediates sumoylation, the effects of the *siz2Δ* mutation are likely indirect in which loss of Siz2 activity causes increased activity by Mms21 independently of the effects of the *slx5Δ* mutation. These findings thus reveal an intricate relationship between Slx5, Esc2 and various SUMO ligases towards sumoylation homeostasis in cells.

### Protein sumoylation has an important role in suppressing duplication-mediated GCRs

Many genes suppress GCRs, and a number of pathways are specific to or have more important roles in suppressing GCRs formed by NAHR between repetitive sequences [4], [5], [12], [62]. Previously several genes involved in sumoylation, *ESC2*, *SLX5*, and *SLX8* (Putnam et al. 2009), were shown to play important roles in suppression of duplication-mediated GCRs, whereas *SIZ1* was shown to play little or no role [12], [13]. Here we have extensively examined interactions between genes involved in SUMO homeostasis, which in combination with identification of sumoylated targets specific to each E3 SUMO ligase provides some insight into roles of proteins sumoylation in maintenance of genome stability.

Mutations in *ESC2* and *MMS21* give rise to substantially greater defects in the duplication-mediated GCR assay than in the assay in which GCRs are formed by single copy sequences. Epistasis between SUMO E3 ligase domain mutations in *MMS21* and *ESC2* indicate that both genes acting the same pathway, although deletion of *ESC2* appears to have a weaker phenotype in most assays than *mms21-11* or *mms21-CH*. This epistasis is consistent with the fact that both genes suppress X-shaped DNA replication intermediates [7], [39], [40] and both promote sumoylation of the same targets (Figure 4), including the SMC-family of proteins and proteins in the nucleolus. In contrast, deletion of *SIZ1* or *SIZ2* causes much smaller effects, with the deletion of *SIZ2* causing a greater defect in genome stability when combined with mutations of either *MMS21* or *SLX5*. Together, these results suggest that an Mms21- and Esc2- specific sumoylation event plays a crucial role in suppressing duplication-mediated GCR formation.

Increases in the rate of duplication-mediated GCRs could arise through increased levels of DNA damage, increases in the overall levels of HR, and/or defects in suppressing NAHR relative to HR. Physical restraints between sister chromatids via cohesin or the damage-induced Smc5-Smc6 complex allows for appropriate repair. Consistent with this view, a single double-stranded break is sufficient to establish genome-wide sister chromatid cohesion even on undamaged chromosomes independent of DNA replication [63], [64]; cohesin mutants are sensitive to ionizing radiation [65]; and the DNA damage checkpoint also mediate the stability of the securin Pds1 to prevent sister chromatid separation [66]–[69]. Moreover, sumoylation of the cohesin subunit Mcd1 by Mms21 is induced by a double strand break and is required for establishing cohesion between sister chromatids both at the double-strand break and in the genome-wide response [28]. Consistent with the remarkable defects observed by mutation of *MMS21* or *ESC2* but not *SIZ1* or *SIZ2*, we hypothesize that modification of Mms21-specific targets, most likely SMC-family proteins and/or associated proteins observed here, are required to maintain sister chromatid cohesion and prevent NAHR.

Remarkably, deletion of *SLX5* and *SLX8* also cause increased rates of duplication-mediated rearrangements (Table 1, Putnam et al., 2009), despite the fact that deletion of *SLX5* causes an increase in the sumoylation of Mms21-targets. Moreover, the deletion of *SIZ2*, which also increases the sumoylation of Mms21-targets, causes a greater defect in suppressing duplication-mediated rearrangements alone and in combination with *esc2Δ*, *slx5Δ*, *mms21-11*, and *mms21-CH* than deletion of *SIZ1*, which does not affect Mms21-targets (Table 1). Together these data would suggest that careful maintenance of the sumoylation levels of Mms21 targets is required to maintain genomic stability or that deletion of *SLX5*, *SLX8*, and *SIZ2* cause other defects leading to NAHR independent of how *MMS21* suppresses genome stability.

Analysis of the synthetic interactions between mutations affecting viability, target sumoylation levels, or changes in genome stability suggests a mechanism whereby sumoylation by *MMS21* functions in parallel to a pathway comprised of the partially redundant *SIZ1* and *SIZ2* genes (Figure 5C). *ESC2* promotes the accumulation of sumoylated Mms21 targets, likely by recruiting Ubc9 or stabilizing the Mms21-Ubc9 complex, and the inability to generate the *esc2Δ siz1Δ siz2Δ* strain likely corresponds to a substantial loss of the sumoylated proteome. *ESC2* and *SLX5* act in opposing manners to regulate Mms21 activity towards a proper balance of protein sumoylation in cells, which is critical to prevent aberrant genome rearrangements driven by homeologous DNA sequences in the complex genomes of higher eukaryotes.

## Materials and Methods

### Yeast genetic methods

Standard yeast genetics technique was used to generate mutants listed in Table S13. Although no effect on GCRs was observed, 2-micron plasmid was eliminated in SUMO ligase-null mutants using a method described previously [56]. Method for GCR analysis was described previously [70].

### Biochemical methods

To purify sumoylated proteins from HF-SUMO cells, equal amounts of cells from two difference cells, i.e., untagged versus HF-SUMO or wild-type versus SUMO ligase-null mutant, were first combined and lysed together. To identify sumoylated proteins, two replicate experiments were performed to compare untagged cells and HF-SUMO cells. The stable isotopes used were switched in these two replicate experiments. For all other comparisons between wild-type and SUMO ligase-null mutant, wild-type cells were always grown in the light Lys/Arg containing media, while the ligase-null mutant was grown in the heavy Lys/Arg containing media. The combined cells were broken using glass beads in a solution containing 0.2 M sodium hydroxide and 2% SDS. Following cell lysis, 0.2 M hydrochloric acid and 0.1 M Tris pH8.0 buffer was added to adjust the pH of sample to be 8.0. The sample was then heated to 100°C in the presence of 20 mM DTT to reduce proteins, cooled to room temperature and then 50 mM iodoacetamide was added to alkylate free cysteines. The purification of HF-SUMO was done using first Ni-NTA resins and then anti-FLAG resins essentially as described previously [31], [44]. To elute sumoylated proteins from anti-FLAG affinity column, 1 microgram of recombinant Ulp1 catalytic domain was used to cleave the isopeptide bond between sumoylated proteins and HF-SUMO [20]. The Ulp1-eluted sample was then subjected to trypsin digestion for MS analysis [43].

For Western blot analysis to evaluate sumoylation of 3×HA-tagged proteins, each tagged yeast strain was lysed using the same method as above. Cell lysate was then incubated with anti-HA resins, washed and probed by either anti-HA antibody for loading control or anti-FLAG antibody to detect the sumoylated form of the protein.

### MS and data analysis methods

The proteomic method used was described previously [43]. Briefly, tryptic peptides were first fractionated using a HILIC column to generate 10 fractions. Each fraction was then analyzed by LC-MS/MS on an Orbitrap-LTQ MS system. MS data was searched using SEQUEST on a Sorcerer system and quantified using XPRESS as described previously [42]. For database searching, differential modifications of lysine and arginine were included. Each protein was quantified using at least three unique peptides, allowing us to calculate its abundance ratio based on the median and average of these ratios. The identification of SUMO targets further requires them being identified from two replicate experiments using a 10-fold ratio between mock and HF-SUMO as a cutoff (Tables S1 and S2). In cases where a peptide was not identified in the mock sample, the noise level (minimal ion intensity is set to be 1×10^3^ if noise peaks were not detected) in the MS range of the expected peptide was used to calculate the abundance ratio. We then queried these SUMO targets against the results of comparisons between wild-type and each SUMO ligase-null mutant to obtain the abundance ratio for each SUMO target. For protein quantification, the relative abundances of multiple peptides of each SUMO target were used to calculate its abundance ratio based on the median of all ratios. The use of multiple peptides per protein allows an accurate measurement of its abundance change. Occasionally, some SUMO targets were not identified in all the MS experiments, which are indicated in Figure 2 and Supplementary Tables. The same methods were used to analyze the relative abundance of SUMO targets between wild-type and various mutants (see text for details).

## Supporting Information

Figure S1 Patch analysis of the roles of Siz1, Siz2, Mms21, Esc2 and Slx5 in suppressing duplication-mediated GCRs. A) Patch analysis of *siz1Δ*, *siz2Δ* and *siz1Δ siz2Δ* mutants in duplication-mediated GCR strain background. B) Patch analysis of *mms21-11* mutant in either duplication or non-duplication mediated GCR strains. C) Patch analysis of *mms21-11* and *esc2Δ* mutants in the duplication-mediated GCR strain. D) Patch analysis of *slx5Δ*, *siz1Δ* and *siz2Δ* mutants in the duplication-mediated GCR strain.
(PDF)

Figure S2 A) Comparison between the proteins identified in previous proteomic studies [32]–[35]. B) Comparison between this study and each of the other four proteomic studies shows a greater overlap between this study and those of Wohlschlegal *et al* and Denison *et al*. The average numbers of peptides identified per protein in these studies are indicated, along with the average abundance ratios of proteins detected in this study.
(PDF)

Figure S3 Quantification of the relative abundance of SUMO targets between *esc2Δ* and *siz1Δ siz2Δ* mutants, using results from Tables S5 and S8.
(PDF)

Figure S4 Comparison of the relative abundance of SUMO targets between wild-type and *siz1Δ slx5Δ* (black bars) and that between wild-type and *siz1Δ* mutant (grey bars), using results from Tables S3 and S11.
(PDF)

Table S1 Detailed MS data for the comparison between mock (isotopically light) and HF-SUMO (isotopically heavy) cells, including peptide identified, name of the proteins and their abundance ratios.
(XLSX)

Table S2 Detailed MS data for the comparison between HF-SUMO cells (isotopically light) and mock (isotopically heavy), including peptide identified, name of the proteins and their abundance ratios.
(XLSX)

Table S3 Detailed MS data for the comparison between WT and *siz1Δ* mutant, including peptide identified, name of the proteins and their abundance ratios.
(XLSX)

Table S4 Detailed MS data for the comparison between WT and *siz2Δ* mutant, including peptide identified, name of the proteins and their abundance ratios.
(XLSX)

Table S5 Detailed MS data for the comparison between WT and *siz1Δ siz2Δ* mutant, including peptide identified, name of the proteins and their abundance ratios.
(XLSX)

Table S6 Detailed MS data for the comparison between WT and *mms21-11* mutant, including peptide identified, name of the proteins and their abundance ratios.
(XLSX)

Table S7 Detailed MS data for the comparison between *mms21-11* and *siz1Δ siz2Δ* mutant, including peptide identified, name of the proteins and their abundance ratios.
(XLSX)

Table S8 Detailed MS data for the comparison between WT and *esc2Δ* mutant, including peptide identified, name of the proteins and their abundance ratios.
(XLSX)

Table S9 Detailed MS data for the comparison between *mms21-11* and *esc2Δ* mutant, including peptide identified, name of the proteins and their abundance ratios.
(XLSX)

Table S10 Detailed MS data for the comparison between WT and *slx5Δ* mutant, including peptide identified, name of the proteins and their abundance ratios.
(XLSX)

Table S11 Detailed MS data for the comparison between WT and *slx5Δ siz1Δ* mutant, including peptide identified, name of the proteins and their abundance ratios.
(XLSX)

Table S12 Detailed MS data for the comparison between WT and *slx5Δ siz2Δ* mutant, including peptide identified, name of the proteins and their abundance ratios.
(XLSX)

Table S13 Yeast strains used in this study.
(DOCX)
