# Correction: Distinct SUMO Ligases Cooperate with Esc2 and Slx5 to Suppress Duplication-Mediated Genome Rearrangements

**DOI:** 10.1371/journal.pgen.1006302

**Published:** 2016-08-31

**Authors:** Claudio P. Albuquerque, Guoliang Wang, Nancy S. Lee, Richard D. Kolodner, Christopher D. Putnam, Huilin Zhou

S4 Table is a duplicate of S5 Table. The correct S4 Table can be viewed here.

## Supporting Information

S4 Table Detailed MS data for the comparison between WT and *siz2Δ* mutant, including peptide identified, name of the proteins and their abundance ratios. (XLSX)
