# Supplementary figures and images for: Distinct SUMO Ligases Cooperate with Esc2 and Slx5 to Suppress Duplication-Mediated Genome Rearrangements
Source: PLoS Genet. 2013 Aug 1;9(8):e1003670. doi: 10.1371/journal.pgen.1003670 (PMC3731205; doi:10.1371/journal.pgen.1003670)

A)

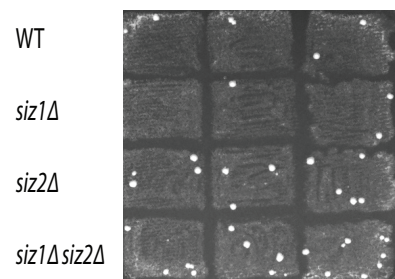

B)

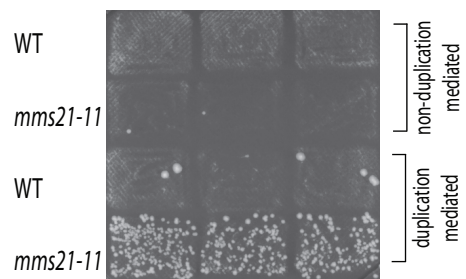

C)

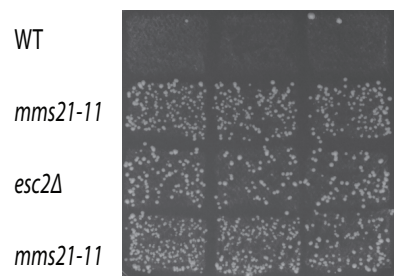

D)

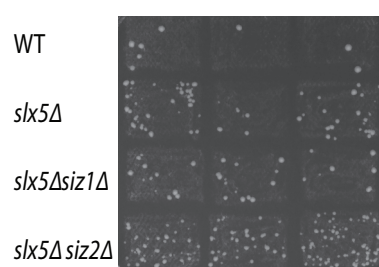

Supplement: Figure S1 — Patch analysis of the roles of Siz1, Siz2, Mms21, Esc2 and Slx5 in suppressing duplication-mediated GCRs. A) Patch analysis of siz1Δ, siz2Δ and siz1Δ siz2Δ mutants in duplication-mediated GCR strain background. B) Patch analysis of mms21-11 mutant in either duplication or non-duplication mediated GCR strains. C) Patch analysis of mms21-11 and esc2Δ mutants in the duplication-mediated GCR strain. D) Patch analysis of slx5Δ, siz1Δ and siz2Δ mutants in the duplication-mediated GCR strain. (PDF) [file pgen.1003670.s001.pdf]

A)

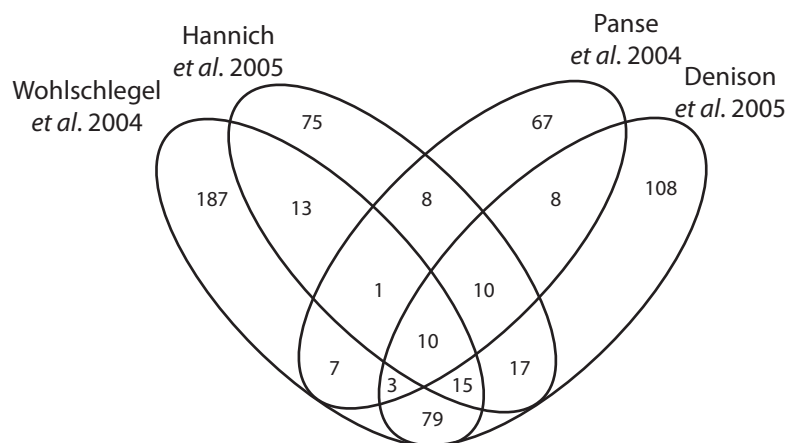

B)

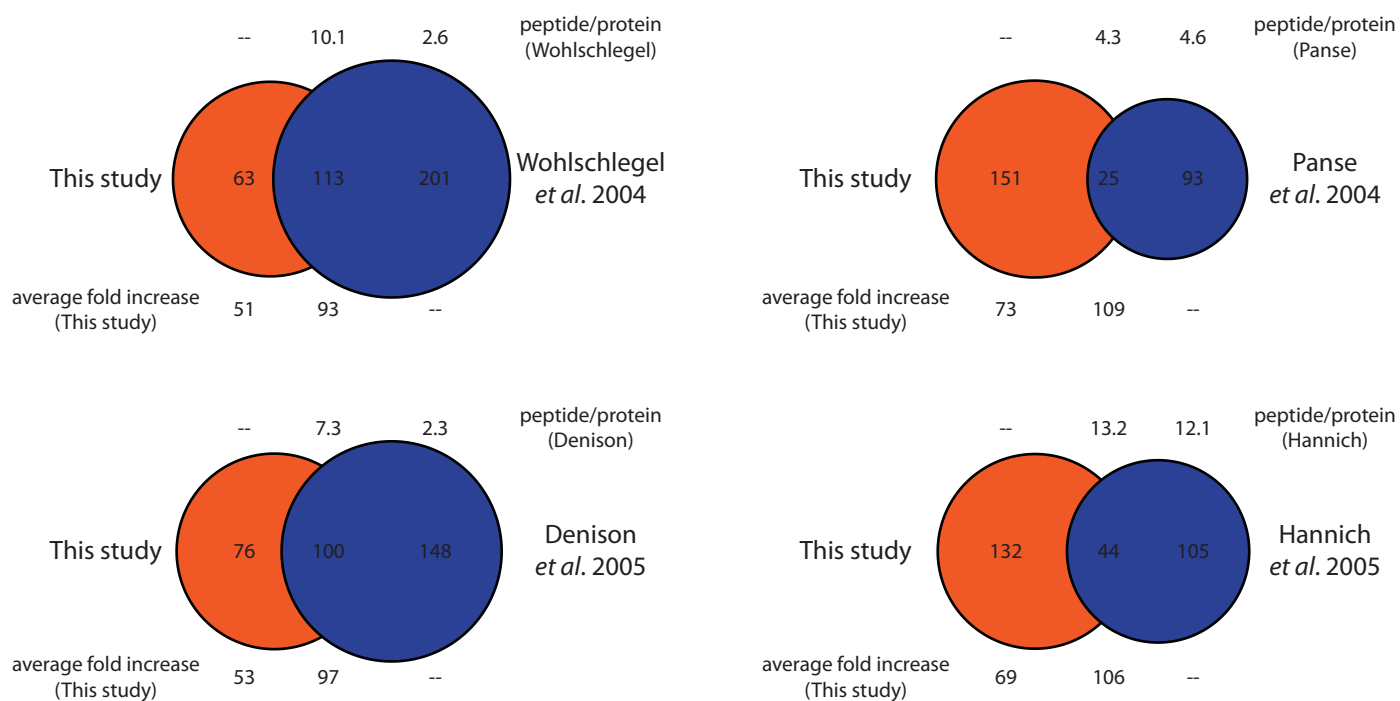

Supplement: Figure S2 — A) Comparison between the proteins identified in previous proteomic studies [32]–[35]. B) Comparison between this study and each of the other four proteomic studies shows a greater overlap between this study and those of Wohlschlegal et al and Denison et al. The average numbers of peptides identified per protein in these studies are indicated, along with the average abundance ratios of proteins detected in this study. (PDF) [file pgen.1003670.s002.pdf]

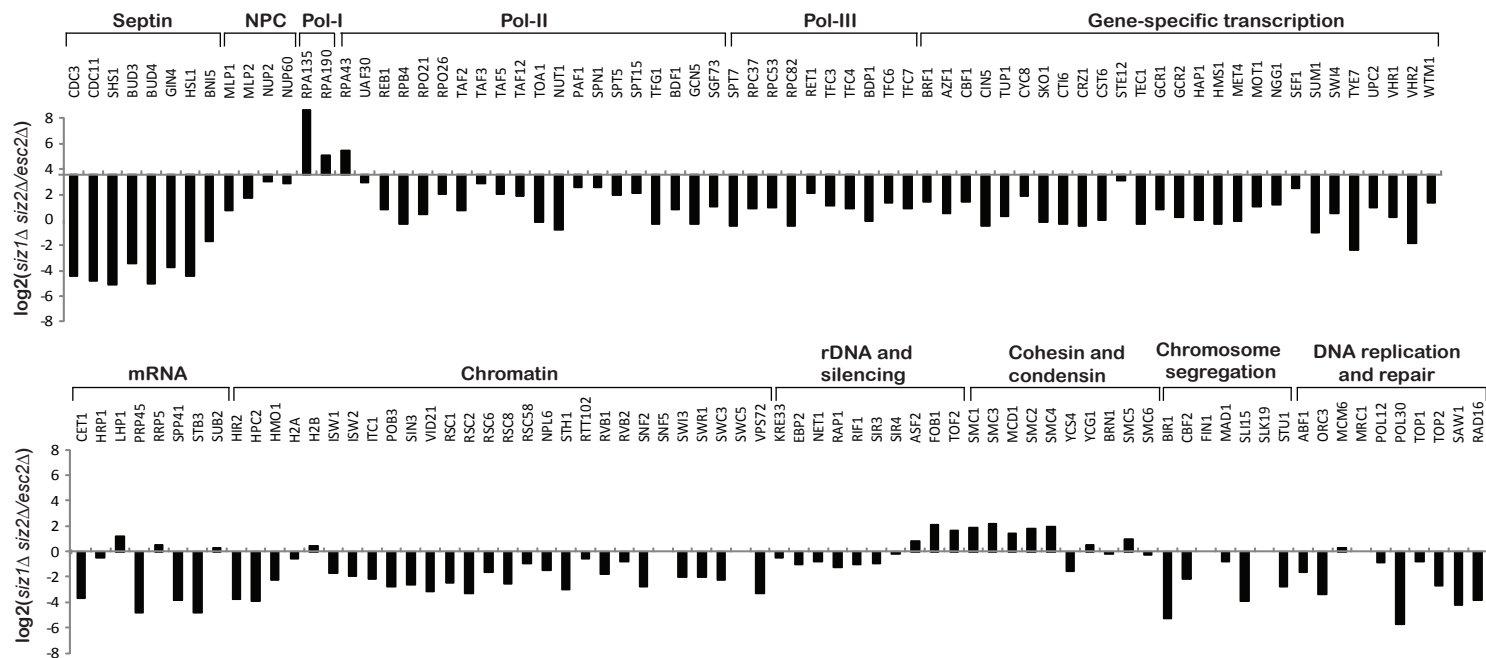

Supplement: Figure S3 — Quantification of the relative abundance of SUMO targets between esc2Δ and siz1Δ siz2Δ mutants, using results from Tables S5 and S8. (PDF) [file pgen.1003670.s003.pdf]

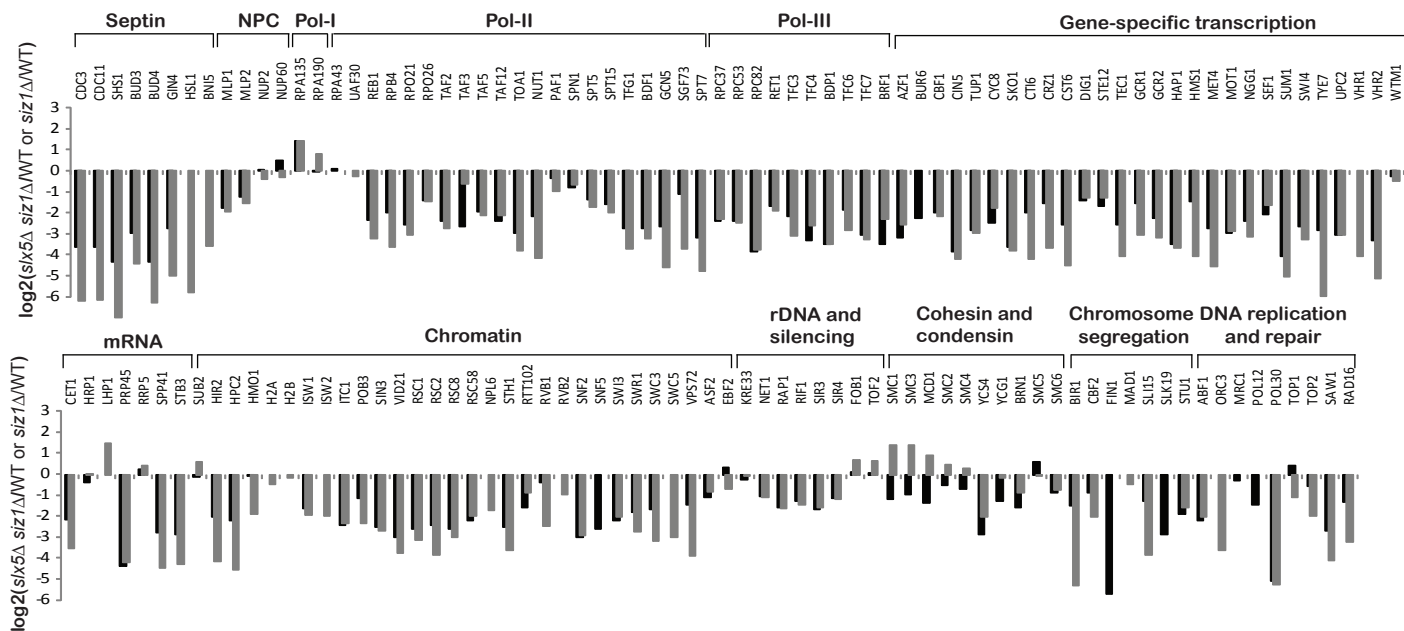

Supplement: Figure S4 — Comparison of the relative abundance of SUMO targets between wild-type and siz1Δ slx5Δ (black bars) and that between wild-type and siz1Δ mutant (grey bars), using results from Tables S3 and S11. (PDF) [file pgen.1003670.s004.pdf]
