# Supplementary material for: Distinct SUMO Ligases Cooperate with Esc2 and Slx5 to Suppress Duplication-Mediated Genome Rearrangements
Source: PLoS Genet. 2013 Aug 1;9(8):e1003670. doi: 10.1371/journal.pgen.1003670 (PMC3731205; doi:10.1371/journal.pgen.1003670)
Supplement: Table S13 — Yeast strains used in this study. (DOCX) [file pgen.1003670.s017.docx]

**Supplementary Table 13.** Yeast strained used in this study.

| RDKY6677 | Putnam et al, Nature 2009 |
| --- | --- |
| RDKY6678 | Putnam et al, Nature 2009 |
| HZY2020 | *siz1∆::*HIS in RDKY6678 |
| HZY2021 | *siz2∆::*HIS in RDKY6678 |
| HZY2035 | *siz1∆::HIS siz2∆::*NAT in RDKY6678 |
| HZY2047 | *mms21-11::G418* in RDKY6678 |
| HZY2148 | *mms21-11::G418* *siz1∆::*HIS in RDKY6678 |
| HZY2149 | *mms21-11::G418* *siz2∆::*NAT in RDKY6678 |
| HZY2145 | *mms21-CH::G418* in RDKY6678 |
| HZY2173 | *mms21-CH::G418* in RDKY6677 |
| HZY2356 | *mms21-CH::G418 siz1∆::*HIS in RDKY6678 |
| HZY2370 | *mms21-CH::G418 siz1∆::*HIS in RDKY6677 |
| HZY2372 | *mms21-CH::G418 siz2∆::*HIS in RDKY6678 |
| HZY2435 | *mms21-CH::G418 siz2∆::*HIS in RDKY6677 |
| HZY2082 | *siz2∆::*HIS in RDKY6677 |
| HZY2098 | *siz1∆::*HIS *siz2∆::*NAT in RDKY6677 |
| HZY2086 | *mms21-11::G418* in RDKY6677 |
| HZY2190 | *mms21-11::G418* *siz1∆::*HIS in RDKY6677 |
| HZY2192 | *mms21-11::G418* *siz2∆::*NAT in RDKY6677 |
| HZY2195 | *mms21-11::G418* *esc2∆::*NAT in RDKY6678 |
| HZY2197 | *mms21-11::G418* *esc2∆::*NAT in RDKY6677 |
| HZY2030 | *esc2∆::*NAT in RDKY6678 |
| HZY2084 | *esc2∆::*NAT in RDKY6677 |
| HZY2123 | *esc2∆::*NAT *siz1∆::*HIS in RDKY6678 |
| HZY2150 | *esc2∆::*G418 *siz1∆::*HIS in RDKY6677 |
| HZY2124 | *esc2∆::*NAT *siz2∆::*HIS in RDKY6678 |
| HZY2152 | *esc2∆::*G418 *siz2∆::*HIS in RDKY6677 |
| HZY2125 | *slx5∆::*NAT *siz1∆::HIS* in RDKY6678 |
| HZY2362 | *slx5∆::*G418 *siz1∆::HIS* in RDKY6677 |
| HZY2241 | *slx5∆::*G418 *siz2∆::NAT* in RDKY6678 |
| HZY2365 | *slx5∆::*G418 *siz2∆::NAT* in RDKY6677 |
| HZY2243 | *slx5∆::*G418 *siz1∆::HIS siz2∆::NAT* in RDKY6678 |
| HZY2293 | *slx5∆::*G418 *siz1∆::HIS siz2∆::NAT* in RDKY6677 |
| SCY249 | Chen et al, JBC 2010 |
| HZY2007 | HF-SUMO in SCY249 |
| HZY2061  HZy20 | *siz1∆::*HIS in HZY2007 |
| HZY2062 | *siz2∆::*HIS in HZY2007 |
| HZY2109 | *siz1∆::*HIS *siz1∆::*URA in HZY2007 |
| HZY2106 | *esc2∆::HIS* in HZY2007 |
| HZY2107 | *mms21-11::HIS* in HZY2007 |
| HZY2234 | Vps72-3HA::G418 in HZY2007 |
| HZY2235 | Vps72-3HA::G418, *mms21-11:*:URA in HZY2007 |
| HZY2236 | Vps72-3HA::G418 in HZY2109 |
| HZY2270 | Hpc2-3HA::G418 in HZY2007 |
| HZY2271 | Hpc2-3HA::G418, *mms21-11:*:URA in HZY2007 |
| HZY2272 | Hpc2-3HA::G418 in HZY2109 |
| HZY2273 | Rap1-3HA::G418 in HZY2007 |
| HZY2204 | Smc1-3HA::G418 in HZY2007 |
| HZY2205 | Smc2-3HA::G418 in HZY2007 |
| HZY2206 | Smc3-3HA::G418 in HZY2007 |
| HZY2207 | Smc4-3HA::G418 in HZY2007 |
| HZY2220 | Smc1-3HA::G418 in HZY2109 |
| HZY2221 | Smc2-3HA::G418 in HZY2109 |
| HZY2222 | Smc3-3HA::G418 in HZY2109 |
| HZY2223 | Smc4-3HA::G418 in HZY2109 |
| HZY2224 | Ycs4-3HA::G418 in HZY2007 |
| HZY2225 | Ycs4-3HA::G418 in HZY2109 |
| HZY2226 | Ycs4-3HA::G418, *mms21-11::*URA in HZY2007 |
| HZY2227 | Brn1-3HA::G418 in HZY2007 |
| HZY2228 | Brn1-3HA::G418 in HZY2109 |
| HZY2229 | Brn1-3HA::G418, *mms21-11::*HIS in HZY2007 |
| HZY2230 | Smc1-3HA::G418, *mms21-11::*URA in HZY2007 |
| HZY2231 | Smc2-3HA::G418, *mms21-11::*URA in HZY2007 |
| HZY2232 | Smc3-3HA::G418, *mms21-11::*URA in HZY2007 |
| HZY2233 | Smc4-3HA::G418, *mms21-11::*URA in HZY2007 |
| HZY2201 | Smc2-3HA::G418 in HZY2106 |
| HZY2202 | Smc4-3HA::G418 in HZY2106 |
| HZY2276 | Rpa135-3HA::G418 in HZY2007 |
| HZY2277 | Rpa135-3HA::G418 in HZY2109 |
| HZY2278 | Rpa135-3HA::G418, *esc2Δ::*URA in HZY2007 |
| HZY2279 | Rpa135-3HA::G418, *mms21-11::*URA in HZY2007 |
| HZY2108 | *slx5∆::*HIS in HZY2007 |
| HZY2113 | *slx5∆::*NAT *siz1Δ::*HIS in HZY2007 |
| HZY2118 | *slx5∆::*NAT *siz2Δ::*HIS in HZY2007 |

**References:**

Denison, C., Rudner, A.D., Gerber, S.A., Bakalarski, C.E., Moazed, D., and Gygi, S.P. (2005). A proteomic strategy for gaining insights into protein sumoylation in yeast. Mol. Cell Proteomics *4*, 246–254.

Hannich, J.T., Lewis, A., Kroetz, M.B., Li, S.-J., Heide, H., Emili, A., and Hochstrasser, M. (2005). Defining the SUMO-modified proteome by multiple approaches in Saccharomyces cerevisiae. J. Biol. Chem. *280*, 4102–4110.

Panse, V.G., Hardeland, U., Werner, T., Kuster, B., and Hurt, E. (2004). A proteome-wide approach identifies sumoylated substrate proteins in yeast. J. Biol. Chem. *279*, 41346–41351.

Wohlschlegel, J.A., Johnson, E.S., Reed, S.I., and Yates, J.R. (2004). Global analysis of protein sumoylation in Saccharomyces cerevisiae. J. Biol. Chem. *279*, 45662–45668.
